# A Case of Carbamazepine-Induced Acute Pancreatitis: A Rare Etiology

**DOI:** 10.7759/cureus.15199

**Published:** 2021-05-23

**Authors:** Asim Ali, Gibson O Anugwom, Warda Naqvi, Mohammad Omar Saeeduddin, Romil Singh

**Affiliations:** 1 Internal Medicine, Hayatabad Medical Complex, Peshawar, PAK; 2 Psychiatry and Behavioral Sciences, West Oaks Hospital, Houston, USA; 3 Psychiatry and Behavioral Sciences, Houston Behavioral Healthcare Hospital, Houston, USA; 4 Infectious Diseases, Shifa International Hospital, Islamabad, PAK; 5 Psychiatry, Liaquat National Hospital and Medical College, Karachi, PAK; 6 Critical Care, Mayo Clinic, Rochester, USA

**Keywords:** carbamazepine, anticonvulsant, acute pancreatitis, trigeminal neuralgia, severe pancreatitis

## Abstract

Carbamazepine-induced acute pancreatitis is rarely reported in the literature. A 49-year-old female presented with sudden onset of severe epigastric pain radiating to the back for the last five hours associated with nausea and two episodes of vomiting. She had been taking carbamazepine for trigeminal neuralgia for the last four weeks. On clinical examination, she was afebrile and had mild tenderness in the epigastrium. Serum chemistry revealed elevated levels of amylase, lipase, and total bilirubin. Her lipid profile was normal, and her abdominal ultrasonography was non-significant. Her abdominal CT revealed generalized pancreatic enlargement with imprecise borders and stranding edema of peripancreatic fat. A possible relationship between carbamazepine and acute pancreatitis was considered due to a lack of other possible causes. Carbamazepine was withdrawn and replaced by oxcarbazepine, and she was managed with bowel rest, isotonic fluids, antiemetics, and analgesics. Her condition improved gradually, and she was symptom-free on day six. She was discharged to her gastroenterology doctor for a follow-up. On her recent visit two weeks later, she was doing well.

## Introduction

Acute pancreatitis is a common cause of hospitalization in the United States, carrying a mortality rate of <1% in mild cases and up to 30% in severe cases of acute pancreatitis [1]. Acute pancreatitis is a medical emergency characterized by inflammation of the pancreas presented with pain in the epigastric region, radiating to the back associated with nausea and vomiting. Multiple conditions can cause acute pancreatitis. Gall stones, alcohol use, and hypertriglyceridemia are among the common causes [2]. Drug-induced acute pancreatitis is rare, counting 0.1-2% of total cases of acute pancreatitis [3]. Several drugs have shown an association with acute pancreatitis in the literature. However, acute pancreatitis induced by carbamazepine is rarely described in the literature [4]. Herein we report a rare case of carbamazepine-induced pancreatitis in a patient with trigeminal neuralgia.

## Case presentation

A 49-year-old female was brought to the ED for severe epigastric pain for the last five hours associated with nausea and vomiting. The pain was sudden in onset, sharp, progressive, and radiating to the back. She had two episodes of vomiting containing food particles, and there was no history of fever, viral infection, acute pancreatitis, alcohol abuse, trauma, travel, and family history of any malignancy. Further history revealed that she had been taking carbamazepine 200mg/day for her trigeminal neuralgia for the last four weeks and reported improvements in her facial pain. On examination, she was in severe distress due to pain, well oriented to time, place, and person. She had a temperature of 98^o^F, blood pressure of 110/75 mmHg, heart rate of 102/minute, and respiratory rate of 25/minute. On abdominal examination, there was mild tenderness in the epigastric region. Bowel sounds were present with no signs of organomegaly. The rest of her clinical examination was non-significant. On initial laboratory investigations, serum lipase and serum amylase were markedly high, and there was also a mild elevation of total bilirubin (Table 1).

**Table 1:** Initial blood workup.

Parameter	Lab value	Reference range
White blood cells	9,100/mm^3^	4,000-11,000
Red blood cells	4.7 million cells/mm^3^	4.35-5.65
Platelet count	275,000/mm^3^	150,000-350,000
Hemoglobin	12.8 g/dL	14-17
Hematocrit	41.2%	41-51
Serum amylase	722 IU/L	30-110
Serum lipase	2407 IU/L	0-160
Aspartate aminotransferase	36 IU/L	8-35
Alanine aminotransferase	34 IU/L	7-35
Total bilirubin	1.9 mg/dL	0.3-1.2
Blood urea nitrogen	18 mg/dL	08-20
Serum creatinine	1.1 mg/dL	0.7-1.2
C-reactive protein	2 mg/dL	<0.2
Erythrocyte sedimentation rate	24	<22
Serum albumin	3.9 mg/dL	3.5-5.5
Sodium	142 mmol/L	136-145
Potassium	3.5 mmol/L	3.5-5.0
Calcium	8.9 mmol/L	9.0-10.5
Chloride	99 mg/dL	98-106

**Figure 1:**
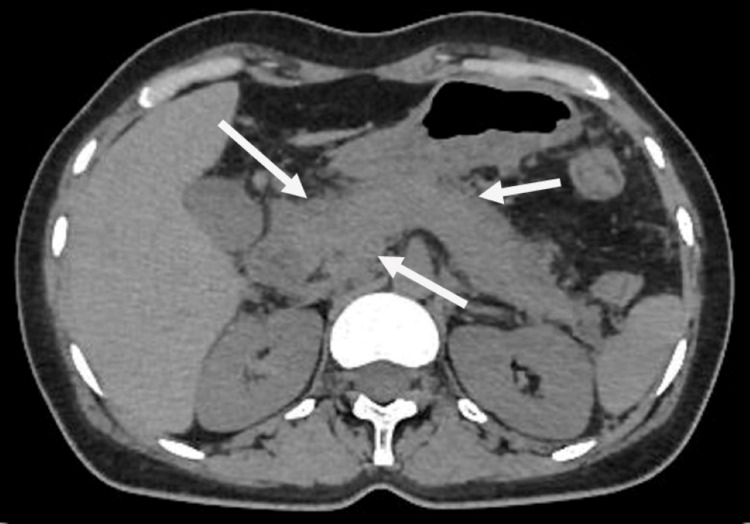
Abdominal CT revealing diffuse enlargement of pancreas with imprecise borders.

Abdominal ultrasonography revealed no abnormalities in the liver, gall bladder, and bile ducts. There was no evidence of cholelithiasis and biliary stone. The lipid profile was within normal range with serum cholesterol of 129 mg/dL, high-density lipoprotein cholesterol (HDL) of 49 mg/dL, low-density lipoprotein cholesterol (LDL) of 51 mg/dL, and serum triglyceride of 131 mg/dL. Abdominal CT was performed, which revealed generalized pancreatic enlargement with imprecise borders and stranding edema of peripancreatic fat (Figure 1). We did not investigate the autoimmune and viral causes of acute pancreatitis due to a lack of suggestive manifestations. Her urine screening was negative for illicit drug use. A possible relationship between carbamazepine and acute pancreatitis was considered due to current therapy and lack of other possible causes. Carbamazepine was withdrawn and replaced by oxcarbazepine on the next day of hospitalization.

**Figure 2:**
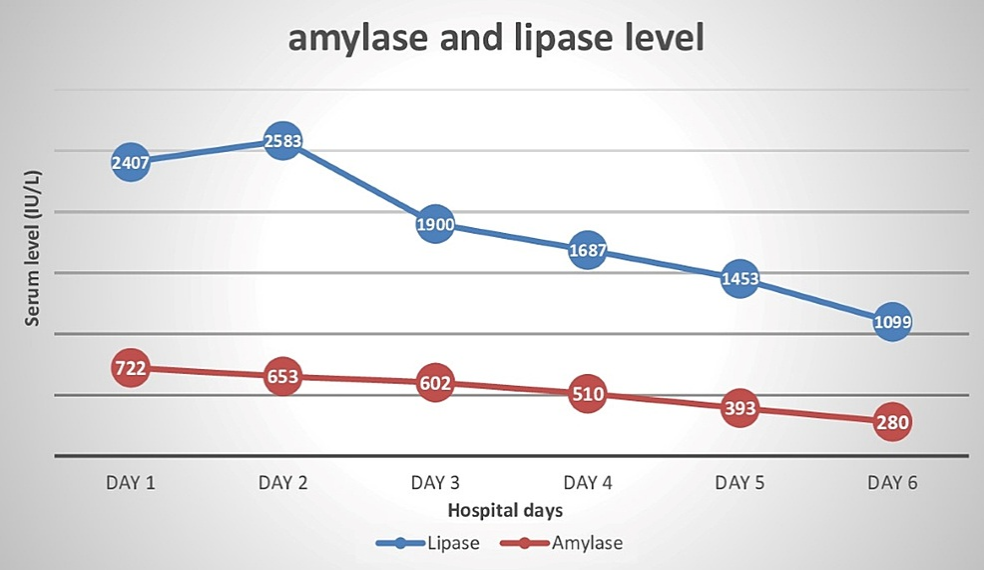
Daily serum lipase and amylase levels during the hospital stay.

She was treated conservatively with bowel rest, proton pump inhibitors, and analgesics. She was resuscitated with isotonic fluids, potassium supplements, and antiemetics in the medical intensive care unit (ICU). Her condition improved on the third day of hospitalization, and daily serum lipase and amylase analysis showed a gradual drop in their levels (Figure 2). The patient was symptom-free on day six, and she started oral nutrition with no aggravation of her signs and symptoms. She was discharged with her gastroenterology doctor for a follow-up on day seven. On her recent visit two weeks later, she was doing well.

## Discussion

Carbamazepine is a common anticonvulsant and has been commonly used to treat trigeminal neuralgia, neuropathic pain, epilepsy, and bipolar disorders. The commonly reported side effects of carbamazepine include nausea, vomiting, dizziness, and constipation, rare cases of allergic reactions, hepatotoxicity, and mood changes [5]. Carbamazepine-induced acute pancreatitis is rarely underlined in the literature. Carbamazepine is considered a Class II medication: medications reported in greater than ten reported cases of acute pancreatitis [4]. Only a small number of acute pancreatitis cases caused by carbamazepine have been reported in the literature [6-8]. Most cases of drug-induced acute pancreatitis usually have a good prognosis and follow a mild or moderate course. However, severe cases can also occur. Causative agents of acute pancreatitis are generally challenging to find. Failure to recognize the offending drug can lead to severe complications and fatal outcomes, including pancreatic pseudocyst, pancreatic necrosis, chronic pancreatitis, and multiorgan failure [9]. Diagnosis of acute pancreatitis is based on clinical presentation, past medical history, clinical examination, serological studies, and imaging modalities. Serum chemistry, including lipase and amylase, are highly specific, and serum levels more than three times the normal value are considered diagnostic in the presence of suggestive clinical presentation. Imaging modalities, including ultrasound abdomen and CT abdomen, confirm the diagnosis and rule out any serious complications of acute pancreatitis [2,10]. However, there are no particular clinical signs and symptoms or laboratory measures specific to drug-induced pancreatitis. Drug-induced pancreatitis can be diagnosed by ruling out all other possible common causes, the occurrence of pancreatitis during medication, resolution of signs and symptoms on withdrawal of the offending agent, and reappearance of manifestations using the same therapy [11]. The precise mechanism of carbamazepine is not well defined. Pathophysiology of drug-induced acute pancreatitis may be idiosyncratic reactions such as pancreatic duct narrowing, hypersensitivity reactions, cytotoxic and metabolic effects, and accumulation of toxic metabolites [3,11]. Acute pancreatitis is managed symptomatically. Treatment includes fluid resuscitation, pain killers, and nutritional provision with enteral or parenteral nutrition if the patient cannot tolerate the oral feeds. The offending drug should be stopped, and alternative medication should be commenced. Antibiotics are usually not recommended in acute pancreatitis [10,12]. Our patient had consistent latency of symptoms onset with the previous cases. She had no history of alcohol use or drug abuse, no evidence of gall stone on abdominal ultrasonography, and the lipid profile was within normal range. Her improvement on withdrawal of carbamazepine and supportive management is likely due to acute pancreatitis caused by carbamazepine.

## Conclusions

Acute pancreatitis is a medical emergency requiring immediate diagnosis and management. Although carbamazepine-induced pancreatitis is rare, it should be considered among differential diagnoses of drug-induced acute pancreatitis. While investigating a case of drug-induced pancreatitis, all other possible causes should be ruled out. A delay in diagnosis can result in adverse outcomes with a prolonged hospital stay. Supportive management and prompt withdrawal of an offending drug can result in improvement and an excellent clinical prognosis.
